# Test-Retest Reliability of a Pendant-Worn Sensor Device in Measuring Chair Rise Performance in Older Persons

**DOI:** 10.3390/s140508705

**Published:** 2014-05-16

**Authors:** Wei Zhang, G. Ruben H. Regterschot, Hana Schaabova, Heribert Baldus, Wiebren Zijlstra

**Affiliations:** 1 Personal Health Solutions Department, Philips Research Europe, High Tech Campus 34, Eindhoven 5656AE, The Netherlands; E-Mail: heribert.baldus@philips.com; 2 University of Groningen, University Medical Center Groningen, Center for Human Movement Sciences, Antonius Deusinglaan 1, Groningen 9713 AV, The Netherlands; E-Mail: g.r.h.regterschot@umcg.nl; 3 Faculty of Biomedical Engineering, Czech Technical University, Nám. Sítná 3105, Kladno 2 272 01, Czech Republic; E-Mail: hankasch@centrum.cz; 4 Institute of Movement and Sport Gerontology, German Sport University Cologne, Am Sportpark Müngersdorf 6, Cologne 50933, Germany; E-Mail: zijlstra@dshs-koeln.de

**Keywords:** chair rise, reliability, fall risk, accelerometer

## Abstract

Chair rise performance is incorporated in clinical assessments to indicate fall risk status in older persons. This study investigated the test-retest reliability of a pendant-sensor-based assessment of chair rise performance. Forty-one older persons (28 females, 13 males, age: 72–94) were assessed in two sessions with 3 to 8 days in between. Repeated chair rise transfers were measured after different instructions. Relative and absolute test-retest reliability of chair rise measurements in individual tests and average over all tests were evaluated by means of intra-class correlation coefficients (ICCs) and standard error of measurement (SEM) as a percentage of the measurement mean. Systematic bias between the measurements in test and retest was examined with paired *t*-tests. Heteroscedasticity of the measurements was visually checked with Bland-Altman plots. In the different test conditions, the ICCs ranged between 0.63 and 0.93, and the SEM% ranged between 5.7% and 21.2%. The relative and absolute reliability of the average over all tests were ICC = 0.86 and SEM% = 9.5% for transfer duration, ICC = 0.93 and SEM% = 9.2% for maximum vertical acceleration, and ICC = 0.89 and SEM% = 10.0% for peak power. The results over all tests indicated that a fall risk assessment application based on pendant-worn-sensor measured chair rise performance in daily life might be feasible.

## 1. Introduction

One in three persons aged 65 or older falls at least once each year [1]. Falls resulting in significant injuries may have huge impact on the lives of the individuals and their families [2]. An early detection of increased fall risk may allow timely interventions and reduce falls or injuries resulting from falls significantly [3]. The clinical assessments of fall risk are usually done using questionnaire or simple field tests [4,5]. Chair rise performance is influenced by the leg strength and power [6,7] and measured as an indicator of fall risk status in old people [8,9]. Field tests with chair rise transfers, such as Timed Up and Go (TUG) and Five Times Sit to Stand (FTSS) are incorporated in fall risk assessments [9]. Single chair rise movement was also used in fall risk assessment tools [5,9]. However, the performance was only evaluated with the time of completing the transfer or subjective evaluation of the difficulty when completing the transfer. The development of on-body sensors in recent years, especially those consisting of inertial sensors like accelerometers and gyroscopes, facilitates new studies and applications in fall risk assessments beyond traditional clinical setup and measurements. Zijlstra *et al.* [10] reported fair to excellent agreements of sit-to-stand (STS) transfer peak power measured with a standard laboratory-based method using force-plate and body-fixed sensors. Other STS transfer measurements using on-body sensors reported in previous studies include duration, velocity, maximum jerk, maximum acceleration and frequency features of accelerations [11–13]. In these studies, measurements were derived from various on-body positions. Measurements from joint estimation of acceleration from chest and thigh fixed sensors were examined in [13]. A sensor fixed at one side of the hip or at the center of mass (COM) was studied in [11,13,14]. In article [10], peak power measured with sensors fixed at the sternum, waist, side of the hip and their combinations were investigated. To better understand the progression of fall risk and provide accurate assessment, prospective longitudinal studies in community settings are needed [2]. In the aforementioned studies, sensor-based analysis was applied in well controlled experiment setups. Sensor fixation and standardized tests with repetition of STS movements restricted the deployment in long-term daily living situations. Hence, the results from previous studies cannot be generalized to daily life situations. A low-cost easy-to-use fall risk assessment tool is desirable from this research perspective.

In our study, we investigate a single sensor-based chair rise transfer analysis solution for continuous fall risk assessment, which is important for monitoring the progress of intervention for fall prevention. To build a power-efficient monitoring and assessment tool, we propose a signal processing method based on one 3D accelerometer instead of methods using power-hungry sensors like gyroscopes. The sensor device can be worn on a necklace in front of the chest. The pendant sensor has the advantage over other body-fixed sensors in terms of convenience and comfort, which can improve user compliance in a long-term measurement and facilitate continuous fall risk assessment in daily living situation. In this article, we report test-retest reliability of a pendant-sensor-based assessment of multiple chair rise measurements in a sample of older persons. Various standardized chair rise tests are performed under the supervision of researchers. Analysis is conducted based on individual tests under different chair rise conditions to demonstrate the reliability of pendant-worn-sensor measurements in a controlled setup. Additionally, analysis based on average measurements over all tests is conducted to understand the reliability when measuring performance of chair rises with larger variance. The latter analysis may give an indication of the measurement reliability in daily living situations, in which various chair rise conditions apply. Hence, the results will give an insight on the feasibility of a fall risk assessment application with chair rise measured in daily life.

## 2. Method

### 2.1. Participants

Subjects were recruited in residential care homes, sheltered houses and health care centers in the Groningen area, The Netherlands. Inclusion criteria were age 70 years or older, ability to stand up from a chair and walk for 10 m at minimum either with or without a walking aid (wheeled walker or cane). Subjects with cardiovascular/respiratory disorders, neurological disorders, severe comorbidity, cognitive disorders that affect comprehension or execution of the physical tests, simultaneous participation in an intervention or exercise program, orthopedic surgery in the previous six months, visual problems to a degree that makes it impossible to walk or stand up safely from a chair, a stroke within the last six months were excluded for recruitment for the study. All subjects signed an informed consent before the start of the study. The study was approved by the Medical Ethical Committee of the University Medical Center Groningen (UMCG), Groningen, The Netherlands.

Forty-one older persons participated in the study. The participants were 28 females and 13 males, age between 72 and 94 years (mean ± SD: 81.9 ± 5.5), body mass ranged between 48 and 104 kg (mean ± SD: 78.5 ± 14), and body height between 1.46 and 1.89 m (mean ± SD: 1.65 ± 0.09). Self-reported number of falls in the year before the study was between 0 and 4. Four participants reported more than one fall in the year before the study. Twelve participants reported one fall in the year before the study.

### 2.2. Study Procedure

The subjects participated in two assessment sessions: test and retest sessions with three to eight days in between. During both assessment sessions, the subjects performed five Sit-to-Stand (STS) tests with comfortable speed from a chair of approximately 0.5 m height. If they experienced no difficulty performing the first five STSs, they were asked to perform another five STSs as fast as possible from the same chair. Each STS test required the subject to start at the sitting position against the back of the chair. After rising up, the subjects stood still for at least five seconds before sitting down to the initial position. After sitting down, they remained in sitting position for at least 10 s before the next transfer started. After completing the STS tests, the subjects performed Sit-to-Walk (STW) tests. They were first asked to perform three rounds of STW with comfortable speed. If they experienced no difficulty performing the first three STWs, they were asked to perform another three rounds of STW tests with fast speed. In each round, the subject was instructed to rise up from the same chair as used for the STS test, walk to a cone, turn around, walk back to the chair and sit down again. At least 10 s resting time was required before the start of the next STW. The subjects practiced STS and STW once before the start of the assessment at each of the two sessions. They were allowed to use their wheeled walker or cane during the assessment if they did so in daily life. In addition, the subjects also performed the standard clinical assessments TUG [15] and FTSS [16]. Three rounds were performed for each test. A researcher recorded the time for completion of each test round.

### 2.3. Data Acquisition

A matchbox-sized hybrid motion sensor device was used for data acquisition. It has a 3D accelerometer with sampling frequency of 50 Hz. The subjects wore the device on a necklace, which was left hanging unrestricted in front of the chest during the assessments. Raw sensor data were recorded and processed offline on a PC.

### 2.4. Data Processing

Raw sensor data were processed and analyzed in MatLab R2012b (The Mathworks, Inc., Natick, MA, United States). The norm (*A_norm_*) of 3D accelerometer data was computed according to Equation (1):

(1) $$A_{\text{norm}}(t)=\sqrt[{2}]{A_{x}{\left(t\right)}^{2}+A_{y}{\left(t\right)}^{2}+A_{z}{\left(t\right)}^{2}}$$

**Figure 1.**
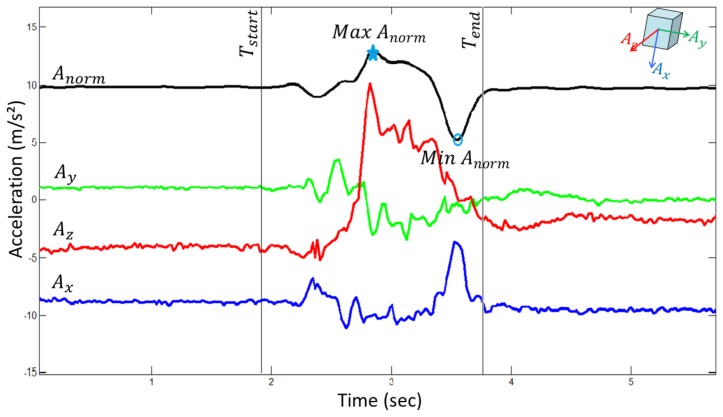
3D acceleration (color) and norm (black) of the acceleration of a chair rise transfer.

A 2nd order Butterworth low-pass filter with cutoff frequency of 3Hz was applied to the norm of acceleration. Figure 1 illustrates the analysis of measurements of a chair rise transfer:

The definitions of the measurements are described below.

#### 2.4.1. Maximum Vertical Acceleration

The measured acceleration on each of the three axes of the pendant-worn-sensor includes a projection of the gravity (always in vertical direction). During a chair rise transfer, the trunk exhibits first a slight forward and downward tilt to position the center of mass from the middle of the chair closer to the feet. The trunk then exhibits an upward movement to lift the body to an upright position till the end of the transfer. In this case, the acceleration measured due to motion is relatively small in magnitude compared to gravity. When computing the norm of the acceleration, the component of the measured acceleration in the horizontal direction (due to trunk tilt and sway) will have a very small contribution to that norm due to the much larger size of gravity, which contributes to the component in the vertical direction. On the other hand, by the same effect, the component of the measured acceleration in the vertical direction will have unaltered contribution to the norm. Based on this assumption, we estimate the vertical acceleration due to the motion of the chair rise transfer as the residual of the norm acceleration (*A_norm_*) subtracting a constant gravity component of 9.8 m/s^2^. The maximum vertical acceleration due to the motion is defined as the residual of the maximum norm acceleration (*Max A_norm_*) minus the gravity.

#### 2.4.2. Duration

The transfer starts at the initiation of the forward and downward trunk rotation before rising up from the chair. It is defined as the last sample when the norm acceleration remains at the gravity level (based on visual selection), which is indicated by the vertical line *T_start_* in Figure 1. The transfer ends when the trunk reaches the upright position. It is defined as the first sample when the norm acceleration reaches the gravity level after the minimum value of the norm (*Min A_norm_*), which is indicated by the vertical line *T_end_* in Figure 1. Duration is the time in seconds between the start and the end of the transfer.

#### 2.4.3. Peak Power

Peak power is the maximum value of the power exertion during the transfer [10]. The computation of power exertion (*P*) during a chair rise transfer is defined in Equation (2), in which *V_vert_* denotes vertical velocity and is computed using Equation (3) (*V_vert_* is zero at the start of the transfer *t_0_*):

(2) $$P(t)=\text{BodyMass}\cdot A_{\text{norm}}(t)\cdot V_{\text{vert}}(t)$$

(3) $$V_{\text{vert}}(t)=V_{\text{vert}}(t_{0})+\int_{t_{0}}^{t}[A_{\text{norm}}(t)-9.8]\cdot \text{dt}$$

#### 2.4.4. Maximum Jerk

Jerk is computed as the 1st derivative of the norm acceleration. Maximum jerk is the maximum value to be found in the interval between the start of the transfer and the time the maximum norm acceleration is reached [12].

To minimize the inter-individual differences due to the differences in body dimensions, all measurements were scaled according to the scaling method introduced in [17]. The follow-up analysis was based on dimensionless pendant-worn-sensor measurements.

### 2.5. Statistical Analysis

All statistical analyses were done in MatLab R2012b (The Mathworks, Inc.). The test-retest reliability was evaluated with the average score of the measurement in each individual test and over all tests. Subjects who completed both test and retest assessment sessions were included for analysis. Subjects with at least two transfers from STS test (normal or fast) and at least two transfers from STW test (normal or fast) successfully recorded were included for analysis. Bland-Altman plots [18] were used to visually check the heteroscedasticity of the data. Paired *t*-test was applied to examine the existence of systematic bias between the measurements in the test and retest [19]. The p-value and the confidence interval (CI) at 95% boundary for the null hypothesis of no difference in means of test and retest data was evaluated. If the p-value is equal or lower than 0.05 or zero value is outside of the CI, a significant difference in means of test and retest measurements is present.

Relative test-retest reliability is the degree to which the individuals maintain their position in a sample over repeated measurements [19]. Relative reliability was evaluated with Intra-Class Coefficient (ICC) model (3, k) defined in [20]. The applied ICC model takes both systematic and random errors in the data into account and uses the mean scores of repeated tests as evaluation score. ICC scores equal or larger than 0.75 are considered to be excellent relative reliability. Fair to good relative reliability is with ICC score in range of 0.4 and 0.75. ICC score lower than 0.4 is considered as poor relative reliability [19].

Absolute test-retest reliability is the degree to which repeated measurements vary for individuals [19]. Absolute test-retest reliability was analyzed with standard error of measurement (SEM) [19] defined in Equation (4), in which SD is the standard deviation of the samples in test and retest. SEM measures the precision of the individual scores on a test. A SEM score in percentage of the mean (SEM%) of test and retest scores is a unit-less value, which is computed with Equation (5) and used in the analysis to compare the absolute test-retest reliabilities of different measurements. A SEM% value equal or smaller than 10% indicates excellent absolute reliability of the measurement:

(4) $$\text{SEM}=\text{SD}\sqrt{1-\text{ICC}}$$

(5) $$\text{SEM}\%=100*\text{SD}\sqrt{1-\text{ICC}}/\text{Mean}$$

Besides the sensor-based chair rise measurements, the test-retest reliabilities of the clinical assessments were also evaluated. The average scores of the three rounds of TUG tests and average scores of the three rounds FTSS tests were computed. Paired t-test was applied for examination of systematic bias in average scores of test and retest sessions. ICC and SEM% of the average scores were computed.

## 3. Results

For the clinical assessments, data from 38 subjects in TUG tests and from 35 subjects in FTSS tests were analyzed. Four subjects in TUG tests and 6 subjects in FTSS tests did not perform the test successfully in at least one of the assessment sessions, thus they were excluded for the analysis. Table 1 summarizes the test-retest reliability evaluation of the clinical assessments. For each assessment, the mean and standard deviation of the average score over all tests in test (Mean ± SD Test) and retest (Mean ± SD Retest) sessions, and the mean and standard deviation of difference (Mean ± SD Diff) between test and retest scores are listed. The p-values computed in paired t-test and its 95% CI, ICC scores to evaluate the relative reliability, and SEM% to evaluate the absolute reliability are shown.

Both TUG and FTSS demonstrated excellent relative and absolute reliabilities. A *p*-value of the paired *t*-test below 0.05 and zero value outside of the 95% CI in average FTSS indicated a significant difference between the mean scores in test and retest sessions. TUG test demonstrated excellent relative and absolute test-retest reliability with ICC of 0.99 and SEM% of 5.4%, respectively. FTSS also had excellent relative and absolute reliability, though the ICC and SEM% values were lower compared to TUG test. The mean completion time of a TUG test and a FTSS test suggested that a large proportion of subjects in our study are at risk of falls [16].

For the pendant-sensor-based chair rise measurements, 36 subjects' data were analyzed. Five subjects' data were excluded from the analysis. Two persons were excluded due to absence at the retest session. One person was excluded due to a technical problem during assessment. Two persons were excluded because less than two transfers in STW test could be recorded.

**Figure 2.**
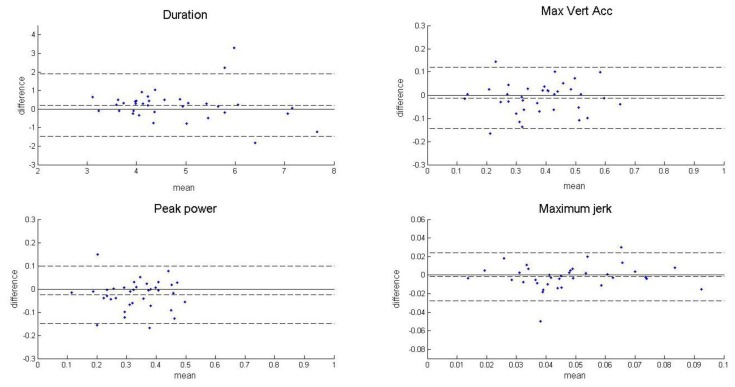
Bland-Altman plots of average measurements over all tests. The mean of differences between the test and the test scores is indicated by the dotted line in the middle of the plot. The upper and the lower boundaries of the 95% CI are indicated by the dotted lines above and below the mean. All measurement scores are dimensionless numbers.

The heteroscedasticity of measurements in each individual and average over all tests was visually examined with Bland-Altman plots. Figure 2 shows the plots of the average scores of the measurements over all tests. The size of the difference between the test and the retest scores did not change with the size of the mean of the test and the retest scores. So, the statistical analysis continued in the original format without logarithmic conversion [18].

Table 2 summarizes the evaluations of test-retest reliability of chair rise measurements in the individual tests. Among the four chair rise tests, the peak power and the maximum jerk had moderate relative reliability in the fast STS test. For the other tests, all measurements demonstrated an excellent relative reliability. An excellent absolute reliability was seen in the transfer duration of the fast STS test and in the transfer duration, the maximum vertical acceleration and the peak power of the fast STW test. Duration, maximum vertical acceleration and peak power demonstrated excellent reliability in most of the chair rise tests and moderate (SEM% less than 15%) to excellent absolute reliability in all the tests. Maximum jerk demonstrated excellent relative reliability with ICC above 0.75 in most tests but worse SEM% scores in all the tests compared to other measurements.

Table 3 summarizes the outcome of the analysis based on average scores of measurements over all tests. The total number of chair rise transfers (in all four tests) recorded in the test session ranged from 7 to 17 (12.83 ± 3.82) and in the retest session from 6 to 17 (12.81 ± 3.82). The average difference in the number of transfers between the test and the retest was −0.03 with a standard deviation of 1.25. The *p*-value of the *t*-test was 0.89, which indicated that no significant difference in means of the number of transfers between the test and the retest assessments. The *p*-values of the duration, the maximum vertical acceleration and the maximum jerk were all greater than 0.05, which indicated no significant difference in means of the test and the retest scores. The peak power had a *p*-value smaller than 0.05 and the lower boundary of the 95% CI was at zero, which indicated a significant difference between the test and the retest scores. All the average scores of measurements over all tests had ICC larger than 0.85. Maximum vertical acceleration had the highest ICC of 0.93 and maximum jerk had the lowest ICC of 0.86. The SEM% indicated excellent absolute reliability in the duration, the maximum vertical acceleration and the peak power but not in the maximum jerk. The maximum vertical acceleration had the lowest SEM% score, hence the highest absolute reliability. The duration, the maximum vertical acceleration and the peak power demonstrated both excellent relative and absolute reliability. The maximum jerk had only excellent relative reliability. Among all the average measurements over all tests, the maximum vertical acceleration had the highest relative and absolute reliability.

## 4. Discussion

In this study, we proposed a chair rise performance analysis solution using a single 3D accelerometer. We anticipated that the convenience and comfort of the pendant-worn-sensor will increase compliance in a long-term fall risk assessment and intervention application in daily life, which is an important factor for maximization of the intervention effects. The chair rise performance analysis based on a single 3D accelerometer developed in our study is an energy-efficient solution compared to methods requiring multiple sensor modalities with gyroscope and magnetometer, which would be another advantage for long-term monitoring applications.

Test-retest reliabilities of the clinical assessments were first evaluated and compared to results in other studies. For the TUG test, excellent relative and absolute reliability was found in 38 older persons. The excellent reliability of TUG test was confirmed by the study [21] with 77 community-dwelling older persons. Excellent relative reliability with ICC = 0.9 and absolute reliability with SEM% = 9% were seen in the FTSS test in our study, which was close to the results reported in the study described in [22] with 29 older females. A significant difference was present between the test and retest FTSS scores in our study, which was not seen in the study presented in [22]. The difference in the test-retest reliability of clinical assessments found in our study and other studies may be due to differences in the total number of subjects and the profiles of the subjects.

To understand the measurement quality of the pendant-worn sensor, relative and absolute test-retest reliability of the measurements of chair rise transfers performed by older persons in various standardized tests were evaluated. Additionally, we analyzed the test-retest reliability of average measurements over all tests for an indication of the measurement quality in daily life.

In the individual tests, excellent relative reliability was demonstrated by most of the measurements except the transfer peak power and the maximum jerk measured in the fast STS test. These results indicated good measurement consistency in repeated chair rise measurements using the pendant-worn sensor in standardized tests. Among all the measurements, maximum vertical acceleration had the highest ICC scores in normal STS, normal and fast STW tests. The maximum vertical acceleration was less influenced by the detection of the start and the end of the transfer. Therefore, less random error was present in this measurement. All the other measurements were estimated based on the detected transfer timing as illustrated in Figure 1. Hence, random error in the timing detection was inherited by the subsequent parameter estimation. The transfer duration, the maximum vertical acceleration and the peak power demonstrated moderate to excellent absolute reliability measured in the fast STS test. The maximum jerk had poorer absolute reliability compared to the other measurements in the individual tests. In study [12], the chair rise transfer peak power measured by a sensor fixed at the right side of the hip showed excellent relative and absolute reliability in both normal and fast STS tests. The study estimated power exertion using hybrid sensor modalities of an accelerometer, a gyroscope and a magnetometer. In our study, the moderate relative and absolute reliability of the peak power in the fast STS test might due to the error in power estimation based on single 3D acceleration signal. Our assumption that a small contribution from the acceleration of horizontal movement to the norm could be neglected might not be true in the fast STS transfers. The horizontal acceleration, especially during the bending forward downward at the beginning, may be influenced by the speed of the transfer. In this case, the accuracy of the vertical acceleration estimation with the norm of the 3D acceleration might be compromised.

All the average measurements over all tests demonstrated excellent relative reliability. The maximum vertical acceleration, the peak power and the maximum jerk had higher ICC scores than those in the individual tests. The transfer duration, the maximum vertical acceleration and the peak power also showed excellent absolute reliability. The maximum jerk had moderate absolute reliability. The absolute reliability in the average measurements over all tests was lower than those measured in the fast STW test, which can be explained by the computation of SEM%. According to Equation (5), SEM% was in a positive association to the ratio of the standard deviation (SD) to the mean of the measurements. The difference in profiles of the subjects in the analysis was reflected in the ratio of SD to mean. Only 16 out of 36 subjects were able to perform the fast STW test during the assessments. Compared to the whole group of subjects, those individuals included in the fast STW test were prone to be physically healthier and with better mobility-related functions. For example, the ratio of SD to mean of maximum vertical acceleration of the subjects in fast STW test was 0.26 (Mean = 0.53, SD = 0.14), while the ratio of SD to mean of all subjects in average over all tests was 0.34 (Mean = 0.38, SD = 0.13). As the relative reliabilities (ICCs) of the measurements in the fast STW test and the average over all tests were similar, larger SEM% was observed in the average over all tests whose SD to mean ratio was larger.

The chair rise transfer duration, the maximum acceleration and the peak power measured with the pendant-worn sensor showed similar reliability compared to the FTSS test, but a less good reliability compared to the TUG test. TUG test involves a small fraction of chair rise and a large fraction of walking, which might explain the relatively bigger discrepancy in reliability analysis when it was compared to only chair rise movements. In terms of the effort of test setup, TUG test requires space in the test environment for straight walking with an accurate measure of 3-meter distance, which may not be realizable in daily living situations, whereas the pendant-worn sensor could be applied with little restriction in test setup. As the standardized clinical assessment implied, that a large population in our study was at risk of falling, the results of pendant-worn-sensor based measurements were therefore representative for the target group. In an earlier study, STS peak power and related measures with the hip-fixed sensor were found to have a higher sensitivity to detect effects of training leg strength, leg power and balance than standard clinical fall risk assessments, such as TUG test [12]. In addition, STS analyses based on sternum and hip fixed sensors have demonstrated fair to excellent linear relationship with the STS peak power measured with the clinical assessment using force plates [10]. Hence, we expect the chair rise measurements with the pendant-worn sensor having similar clinically meaningful outcomes. However, this needs to be confirmed in the future study.

There are some limitations in the current study. A difference in means between the test and the retest was present in the transfer peak power but not in the duration, the maximum vertical acceleration or the maximum jerk. In Figure 2, a small increase of the transfer duration showed in the retest (explained by the position of the dotted line in the middle of the plot). Meanwhile, a small decline in the maximum vertical acceleration showed in the retest. For a chair rise transfer, the power exertion is in a negative association with the duration, whereas in a positive association with the vertical acceleration. The small non-significant bias in the duration and the vertical acceleration may lead to a larger and significant bias after integration to derive power exertion. The importance of the maximum jerk for chair rise performance needs to be defined, which may lead to a better understanding of the moderate absolute reliability in this measurement. In the daily life situation, variance in chair rise transfers may be larger than in the four test conditions covered in our study. For example, rising up from different chairs, which might be an additional variance resource, was not analyzed in this study. In daily life, the pendant sensor might be expected to be worn under the clothes and tilt. The chair rise measurements were estimated based on the norm of acceleration, which is independent from the sensor orientation. Hence, we expect similar reliability in the measurements when the pendant sensor is worn under the clothes. In future studies, the reliability of pendant-sensor-based measurements needs to be confirmed by analysis in chair rise transfers in real daily life conditions.

## 5. Conclusions

In conclusion, the pendant-sensor-based chair rise measurements demonstrated good to excellent relative reliability and moderate to excellent absolute reliability. Among the measurements evaluated, the transfer duration, the maximum vertical acceleration and the peak power were with excellent relative and absolute reliability in average measurements over all tests. Comparable reliability to FTSS test demonstrated the feasibility of using pendant-worn-sensor as an alternative in standard clinical assessment for fall risk. It will ease the assessment setup and analysis. The average measurements over all tests had higher relative and absolute reliability compared to the measurements in most individual tests. This observation indicated that the average performance of chair rise transfers in daily life which are usually in various manners might be reliably measured. Hence, it seems feasible to use the pendant-worn sensor in daily life for assessment of fall risk in older persons and continuously monitoring their progress on the intervention exercise for fall prevention.

## Figures and Tables

**Table 1.** Test-retest reliability of the clinical assessments.

**Clinical Assessment**	**Mean ± SD Test**	**Mean ± SD Retest**	**Mean ± SD Diff**	***p*-Value**	**95% CI of Diff**	**ICC**	**SEM% (%)**
TUG (sec)	15.77 ± 9.36	15.27 ± 9.19	−0.50 ± 1.68	0.08	−1.05–0.05	0.99	5.4
FTSS (sec)	17.36 ± 4.87	16.29±4.68	−1.07 ± 2.92	0.04	−2.07–0.07	0.90	9.0

**Table 2.** Summary of evaluations of the relative and the absolute reliability in chair rise measurements in individual tests.

**Nr Subjects**	**Normal STS**		**Fast STS**		**Normal STW**		**Fast STW**	
			
	**36**		**24**		**36**		**16**	
			
	**ICC**	**SEM%**	**ICC**	**SEM%**	**ICC**	**SEM%**	**ICC**	**SEM%**
Duration	0.77	10.8	0.86	7.5	0.80	12.4	0.90	5.7
Max Vert Acc	0.86	13.0	0.82	11.5	0.91	10.1	0.93	7.2
Peak power	0.85	11.0	0.63	13.8	0.88	10.6	0.88	8.5
Max Jerk	0.78	20.7	0.66	21.2	0.85	15.2	0.86	12.6

**Table 3.** Test-retest reliability analysis based on average scores of measurements over all tests. All measurement scores are dimensionless numbers.

**Measurement**	**Mean ± SD Test**	**Mean ± SD Retest**	**Mean ± SD Diff**	***p*-Value**	**95% CI of Diff**	**ICC**	**SEM% (%)**
Duration	4.88 ± 1.16	4.67 ± 1.27	−0.21 ± 0.85	0.15	−0.50–0.08	0.86	9.5
Max Vert Acc	0.37 ± 0.13	0.38 ± 0.13	0.01 ± 0.07	0.30	−0.01–0.03	0.93	9.2
Peak Power	0.32 ± 0.10	0.35 ± 0.10	0.02 ± 0.06	0.03	0.00–0.05	0.89	10.0
Maximum Jerk	0.05 ± 0.02	0.05 ± 0.02	0.00 ± 0.01	0.43	−0.00–0.01	0.86	14.8
